# Assessing the human factors involved in chest compression with superimposed sustained inflation during neonatal and paediatric resuscitation: A randomized crossover study

**DOI:** 10.1016/j.resplu.2024.100721

**Published:** 2024-07-17

**Authors:** Chelsea M.D. Morin, Brenda H.Y. Law, Jonathan P. Duff, Georg M. Schmölzer

**Affiliations:** a Centre for the Studies of Asphyxia and Resuscitation, Neonatal Research Unit, Royal Alexandra Hospital, Edmonton, Alberta, Canada; b Department of Pediatrics, Faculty of Medicine and Dentistry, University of Alberta, Edmonton, Alberta, Canada

**Keywords:** Newborn, Neonatal resuscitation, Human factors, Chest compression with sustained inflation, Simulation

## Abstract

**Background:**

A new cardiopulmonary resuscitation technique, chest compressions with sustained inflation (CC + SI) might be an alternative to both the neonatal [3:1compressions to ventilations (3:1C:V)] and paediatric [chest compression with asynchronous ventilation (CCaV)] approaches. The human factors associated with this technique are unknown. We aimed to compare the physical, cognitive, and team-based human factors for CC + SI to standard CPR (3:1C:V or CCaV).

**Methods:**

Randomized crossover simulation study including 40 participants on 20 two-person teams. Workload [National Aeronautics and Space Administration Task Load Index (NASA-TLX)], crisis resource management skills (CRM) [Ottawa Global Rating Scale (OGRS)], and debrief analysis were compared.

**Results:**

There was no difference in paired NASA-TLX scores for any dimension between the CC + SI and standard, adjusting for CPR order. There was no difference in CRM scores for CC + SI compared to standard. Participants were less familiar with CC + SI although many found it simpler to perform, better for transitions/switching roles, and better for communication.

**Conclusions:**

The human factors are no more physically or cognitively demanding with CC + SI compared to standard CPR (NASA-TLX and participant debrief) and team performance was no different with CC + SI compared to standard CPR (OGRS score).

## Background

Cardiopulmonary resuscitation (CPR), defined as chest compressions (CC) with or without administration of vasopressor, is uncommon in neonates, performed in only about 1.1% of all neonatal intensive care unit (NICU) admissions.^1^ The American Heart Association (AHA) neonatal resuscitation guidelines recommend a 3:1 CC to ventilation ratio (C:V) with 120 events/min (i.e., CC + ventilations).^2^ In comparison, AHA paediatric resuscitation guidelines recommend 15:2C:V (for two rescuers) prior to establishment of an alternate airway and continuous CC with asynchronous ventilations (CCaV) with 100–120 CC/min and 20–30 ventilations/min after establishment of an alternate airway.^3^ The specific CPR algorithm followed depends on patient age, health care provider comfort, and location of CPR, especially for young infants, as there are no specific guidelines for timing of transition from neonatal to paediatric resuscitation guidelines.^2^, ^3^, ^4^

A newer technique for CPR, called chest compression with sustained inflation (CC + SI) may be beneficial for resuscitation in both neonates and paediatric patients.^5^, ^6^ Studies reported reduced time to return of spontaneous circulation (ROSC) in neonatal piglets^5^, ^7^, ^8^, paediatric piglets^6^, and neonates^9^, ^10^ along with increased survival in neonatal^5^ and paediatric piglets.^6^ With this technique, a sustained high-pressure inflation is provided simultaneously with continuous CC, allowing for passive ventilation with pressure changes caused by compression of the chest and recoil of the chest with each CC.^5^ This technique not only has the potential to improve patient outcomes, but also to simplify resuscitation for neonatal and paediatric patients into a single approach.

In addition to the potential physiological differences of CC + SI versus 3:1C:V and CCaV, the clinical effectiveness of CC + SI might also be influenced by human factors such as physical ergonomics, cognition, and team functioning. Fortunately, the need for CPR is rare. Given the rarity and diversity of CPR events in neonatology, team-based simulations are an effective way evaluate human factors during resuscitation, using repeated, controlled, and standardized scenarios.

We aimed to use standardized team-based simulations to 1) evaluate physical, cognitive, and team-based human factors of CC + SI in neonatal resuscitation teams as compared to standard 3:1 C:V or CCaV, 2) uncover potential barriers for implementing CC + SI within the neonatal clinical environment and 3) compare human factors challenges of a novel CPR technique with different teams and physical environments. We hypothesized that the workload, including physical, cognitive, and team-based human factors of performing CC + SI will be perceived as either equivalent to or less demanding than that of 3:1 C:V or CCaV. We further hypothesized that there will be no significant barriers to implementation of CC + SI within the NICU environment and that there will be some unforeseen human factors challenges with CC + SI within different teams and environments.

## Methods

### Study setting

We conducted a randomized crossover simulation study within two NICU sites at our institution. NICU-1, a 69-bed level three perinatal NICU, adjacent to the high-risk obstetrical unit, specializes in complex and extremely premature neonates, born as early as 22 weeks’ gestation. Health care providers (HCPs) are highly trained in neonatal resuscitation, using 3:1 C:V in the delivery room and the NICU. NICU-2, an 18-bed level four referral centre for patients with complex medical, cardiac, and surgical needs within a children’s hospital. Deliveries are very rare at this site and therefore resuscitation only occurs in the NICU. HCPs are trained in both 3:1 C:V and CCaV (for infants with congenital heart disease or > 44 weeks corrected age) CPR techniques. The study was approved by the Health Ethics Research Board at the University of Alberta (Pro00130209). The study protocol has been uploaded as supplementary file.

### Outcomes

National Aeronautics and Space Administration Task Load Index (NASA-TLX) was used to measure subjective workload for participants in the study. This score includes six dimensions (mental demand, physical demand, temporal demand, performance, effort, and frustration), each scored from 0 to 20. For all dimensions, 0 was considered the lowest level of effort and 20 the highest. A NASA-TLX raw score was calculated multiplying the mean of the scores for each participant in scenario by 5, to create a TLX raw score out of 100.

Simulation videos were reviewed by C.M. and crisis resource management (CRM) performance was scored using the Ottawa Global Rating Scale (OGRS) scoring tool.^11^ With this tool, team performance in each of six categories (overall performance, leadership skills, problem solving skills, situational awareness skills, resource utilization skills, and communication skills) is scored from 1 to 7, with 1 being the worst possible performance and 7 being the best possible performance, for a total score ranging from 7 to 42/42. Debrief sessions were reviewed and transcripts of the sessions recorded by C.M. to extract themes, verbalized challenges, and verbalized suggestions for improvements. Themes were extracted independently by C.M. and B.L. through review of the transcriptions. Any disagreements in themes were discussed until consensus was reached.

### Study participants

Participants were recruited throughout July 2023 based on availability while on-duty at each NICU by C.M. Licenced practical nurses, registered nurses, neonatal nurse practitioners, respiratory therapists, neonatology fellows, clinical associates, and consultants were eligible to participate. Participants were required to have Neonatal Resuscitation Program (NRP) and/or Paediatric Advanced Life Support (PALS) training completed within the last two years. HCPs who did not consent to participate or who had self-reported physical barriers to performing CCs or CPR were excluded. A sample size of 40 participants was selected for the study, including two HCP participants per simulation with 20 total simulations. There are no previous studies to base sample size on, but this sample size was estimated to be sufficient to allow for participation from a variety of disciplines.

### Experimental design and randomization

A two-step computer-generated randomizer (https://www.randomizer.org) was used to randomize the order of the scenarios and the order of resuscitation technique (i.e., CC + SI or 3:1 C:V/CCaV). Block randomization with blocks of four was used. Allocation concealment was achieved using numbered, sealed, opaque envelopes which were opened just before commencing the first of the two scenarios. Blinding of the scenario and intervention to participants and researchers was not possible, as the participants had to perform the intervention for the given scenario and a researcher was running the simulations. Participants were assigned numbers for the post-simulation questionnaire which kept track of which simulation session they were a part of (i.e., participants 1–1 and 1–2 were in in the first session), but not which participant they were within the simulation. Identifying data was removed for analysis to reduce any bias.

After consent was obtained, participants received a five-minute teaching session orienting them to the simulation manikin, explaining CC + SI, and answering participants’ questions. Participants were then given an opportunity to practice CC + SI and their standard method(s) of CPR (i.e., 3:1 C:V and/or CCaV). After this practice session, the participants were randomized and completed the first clinical simulation (scenario 1 and 2 in the Appendix) and CPR technique. If randomized to “standard” resuscitation technique, they could choose to perform either 3:1 C:V or CCaV. In our centre, HCPs will perform either 3:1 C:V or CCaV, depending on which is determined to be more appropriate for a given patient.

During the scenarios, HCPs had to assess the manikin, which became bradycardic (heart rate < 60) or asystolic (as per randomization). HCPs had to perform the CPR technique as per randomization and were asked to switch roles at least once during the resuscitation, as is done in routine clinical care (i.e., every two minutes or whenever fatigued). Following the first scenario, participants were given the second scenario to perform the other CPR technique.

Throughout the simulation, physical exam findings were provided upon request (for example, heart rate or air entry). Participants were allowed to trouble shoot any vital sign changes, but the manikin’s clinical status remained unchanged despite the participants’ interventions. Each simulation was four minutes, to allow for at least one switch of roles, and each participant to perform both CC and airway management. Following the second scenario, participants were asked to complete a survey, including demographic data, two NASA-TLX questionnaires (one for each scenario), and barriers to and ideas for implementing CC + SI. NASA-TLX has strong validity evidence for its ability to assess subjective workload and has successfully been used to assess workload during neonatal resuscitation.^12^, ^13^ This was followed by a semi-structured debrief session based on the Promoting Excellence And Reflective Learning in Simulation debriefing framework, run by a single researcher (C.M.) to discuss any clinical learning points and their thoughts about CC + SI.^14^ If consent was given, video was recorded throughout the simulations and debrief for video analysis.

### Study procedure

**Fig. 1:**
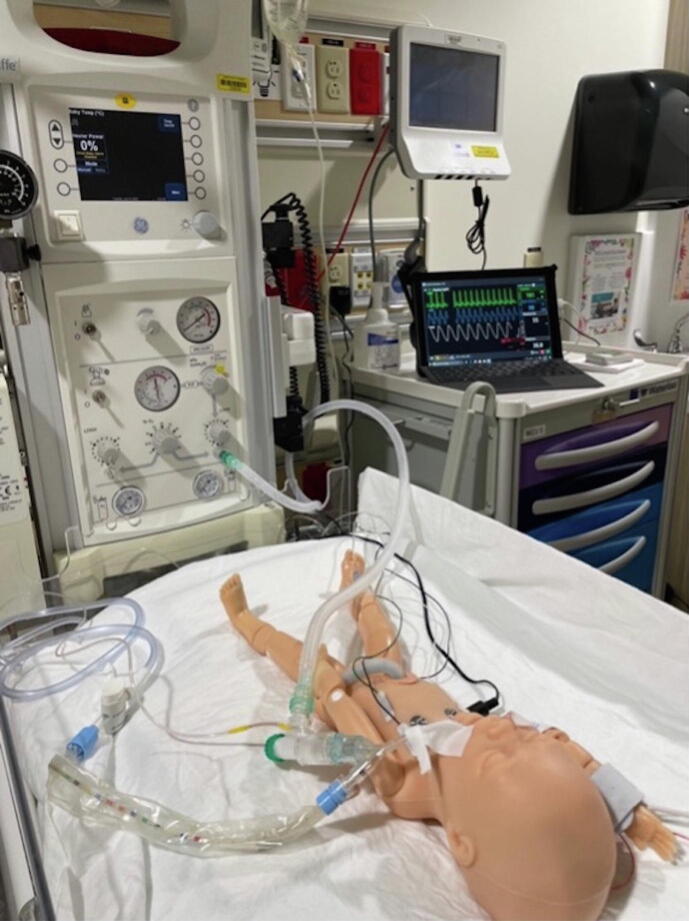
Simulation Setup.

The PremieHal (Gaumard, Miami, USA) simulation manikin was used for each simulation. This manikin has capabilities including the ability to produce visible cyanosis, palpable pulses, and lung and heart sounds that can be auscultated. Monitors (cardiac leads, oxygen saturation sensor, carbon dioxide sensor, and a blood pressure cuff) were connected to the manikin. Simulations were built into Gaumard software and vitals displayed for participants to view during the simulation on a Microsoft Surface laptop (Microsoft, Washington, USA). A picture of the stimulation setup, standardized for all scenarios, is provided in Fig. 1.

### Statistical analysis

Analysis was completed on an intention to treat basis. Data are presented as mean (standard deviation) if normally distributed and median (interquartile range) if skewed. Paired Student’s *t-test* was used for normally distributed data and Wilcoxon matched-pairs signed-rank test for skewed data. A p-value of <0.05 was consider significant. Wilcoxon rank and repeated measures ANOVA were used to account for cross-over effect.

Anonymized survey responses were input into the University of Alberta’s Redcap (Research Electronic Data Capture) database and the data assessed using SAS and STATA® software. The NASA-TLX score includes six dimensions (mental demand, physical demand, temporal demand, performance, effort, and frustration), each scored from 0 to 20. For all dimensions, 0 was considered the lowest level of effort and 20 the highest. A NASA-TLX raw score was calculated multiplying the mean of the scores for each participant in scenario by 5, to create a TLX raw score out of 100.

Simulation videos were reviewed by C.M. and crisis resource management (CRM) performance was scored using the Ottawa Global Rating Scale (OGRS) scoring tool.^11^ With this tool, team performance in each of six categories (overall performance, leadership skills, problem solving skills, situational awareness skills, resource utilization skills, and communication skills) is scored from 1 to 7, with 1 being the worst possible performance and 7 being the best possible performance, for a total score ranging from 7 to 42/42. Debrief sessions were reviewed and transcripts of the sessions recorded by C.M. to extract themes, verbalized challenges, and verbalized suggestions for improvements. Themes were extracted independently by C.M. and B.L. through review of the transcriptions. Any disagreements in themes were discussed until consensus was reached.

## Results

### Quantitative results

Forty HCPs participated including 13 nurses (32.5%), 18 respiratory therapists (45%), 3 neonatal fellows (7.5%), 2 neonatologists (5%), and 4 neonatal nurse practitioners (7.5%), of which 35 (87.5%) identified as female (Table 1). All 40/40 HCPs performed CC + SI, 14/40 performed CCaV, and 26/40 performed 3:1 C:V. All participants completed both simulations, the survey, and the debrief. One debrief session was not recorded as the camera did not record during the session. One student nurse mistakenly participated, which was considered a neonatal nurse for statistical analysis. There was one protocol violation, where one team switched from the assigned CCaV technique to CC + SI during the simulation as they felt their current resuscitation technique was not effective to achieve ROSC. Analysis was completed on an intention to treat basis.

**Table 1:** Participant demographics.

Gender n (%)	Female	Male			
	35 (87.5%)	5 (12.5%)			
Role n (%)	**Registered Nurse**	**Respiratory Therapist**	**Neonatal-Perinatal Medicine Fellow**	**Neonatal-Perinatal Medicine Physician**	**Neonatal Nurse Practitioner**
	13 (32.5%)	18 (45%)	3 (7.5%)	2 (5%)	4 (10%)
Number of times performing CC in the last year (n)	**0**	**1**–**2**	**3**–**4**	**5**–**6**	**7+**
	4	23	3	7	3
Years worked in Current Field	**≤1**	**1.01**–**5**	**5.01**–**10**	**11.01**–**15**	**>15**
	7	13	5	6	9
Intervention performed during resuscitation	**CC + SI**	**3:1C:V**	**CCaV**		
	40	26	14		

Median (IQR) paired TLX raw score was significantly lower for the first simulation compared to the second simulation [first vs second NASA-TLX raw score was 31.5 (21, 45) vs 38.5 (25.5, 50), *p* = 0.04]. Mental and frustration dimension scores were also significantly lower for the first simulation compared to the second simulation (Table 2). Assessing for the crossover impact of the method of CPR performed on the order of CPR, difference in scores for the first simulation compared to the second simulation was only significant for the temporal dimension score (Table 3). There was no significant difference in any of the dimension scores for CC + SI compared with standard CPR in either the first or second simulation independently, or overall, after adjusting for order of CPR (Table 4).

**Table 2:** NASA-TLX scores by order performed and difference in paired scores.

Variable	First score	Second score	Difference in scores	*p*-value
Mental	6 (4,10)	9 (4.5,12)	−0.5 (−3,1)	0.02**
Physical	9 (6,11.5)	10 (5,12)	0 (−1,1)	0.92
Temporal	6.5 (4.5,10)	8 (4.5,10)	−0.5 (−2,1)	0.33
Performance	5 (4,6)	5 (4,8)	0 (−1,0)	0.13
Effort	7 (3.5,10)	9.5 (5.5,12)	0 (−2.5,1)	0.16
Frustration	3.5 (1,8)	5.5 (2,10.5)	0 (−3,0)	0.02**
TLX Raw	31.5 (21,45)	38.5 (25.5,50)	−1 (−12,4)	0.04**

**Significant p-value < 0.05,

Data are presented as median (IQR). P-value given for matched-pairs signed-rank test.

TLX: task load index.

**Table 3:** NASA-TLX difference in paired scores by order performed, crossover analysis.

**TLX Dimension**	**Difference in scores (CC + SI – standard)**		***p*-value**
	Standard^#^ then CC + SI	CC + SI then standard^#^	
Mental	−0.50 (−3.0–1.50)	−1.0 (−4.0–1.0)	0.55
Physical	0.0 (−2.50–2.0)	0.0 (−1.0–1.0)	0.54
Temporal	1.0 (−1.0–2.0)	−1.0 (−2.50–0.0)	0.005**
Performance	0.0 (−1.0–0.50)	0.0 (−2.50–0.0)	0.27
Effort	0.50 (−1.50–2.0)	−2.0 (−3.0–0.0)	0.073
Frustration	−0.50 (−3.0–0.50)	0.0 (−3.0–0.0)	0.85
TLX Raw	1.50 (−12.50–5.0)	−6.50 (−11.50–1.0)	0.30

**Significant p-value < 0.05.

Data are presented as median (IQR). P-value given for Wilcoxon Rank.

Standard^#^: 3:1 chest compression to ventilation ratio or chest compression with asynchronous ventilations.

CC + SI: chest compression with sustained inflation; CPR: cardiopulmonary resuscitation; TLX: task load index.

**Table 4:** NASA-TLX scores by CPR method and order performed.

**Dimension**	**CC + SI score**		^#^**Standard score**		***p*-value**		
	CC + SI first	^#^Standard first	CC + SI first	^#^Standard first	CC + SI vs ^#^standard (first simulation)*	CC + SI vs ^#^standard (second simulation)*	CC + SI vs ^#^standard (adjusted for CPR order)^##,^**
Mental	5.50 (4.0–9.50)	8.50 (3.50–12.50)	8.0 (3.50–10.0)	9.0 (5.0–10.50)	0.37	0.73	0.78
Physical	7.50 (6.0–11.50)	11.0 (5.50–13.50)	10.0 (5.50–11.50)	9.0 (5.0–10.50)	0.53	0.22	0.62
Temporal	5.50 (4.0–7.50)	8.0 (3.50–10.0)	7.0 (4.50–11.0)	8.0 (5.0–10.50)	0.09	0.91	0.19
Performance	5.0 (4.0–6.0)	5.0 (2.50–7.50)	5.0 (3.50–6.0)	5.50 (5.0–8.0)	0.95	0.14	0.26
Effort	7.0 (3.0–10.0)	9.50 (4.0–12.0)	7.50 (4.50–11.50)	9.0 (6.0–10.50)	0.24	0.85	0.50
Frustration	4.50 (1.0–8.50)	5.50 (2.0–11.0)	2.50 (1.0–8.0)	5.50 (2.50–9.50)	0.51	0.90	0.59
TLX raw	31.0 (18.0–42.0)	43.0 (24.50–52.0)	32.0 (21.50–47.0)	38.0 (26.0–47.50)	0.39	0.88	0.67

Data are presented as median (IQR). P-value given for Student’s *t*-test* and repeated-measures ANOVA**.

^#^Standard: either 3:1 chest compression to ventilation ratio or chest compression with asynchronous ventilations.

^##^Of note, unadjusted p-values are all equal to the adjusted p-values to 2 digits.

CC + SI: chest compression with sustained inflation, TLX: task load index.

There was no difference in any of the CRM scores for CC + SI compared to standard CPR (3:1 C:V and CCaV combined), 3:1 C:V alone, or CCaV alone (Table 5).

**Table 5:** Crisis Resource Management scores by CPR method performed and difference in paired scores.

Variable	CC + SI score	Standard^#^ score	Difference in scores	*p*-value		
				CC + SI vs Standard^#^	CC + SI vs 3:1C:V	CC + SI vs CCaV
Overall performance	5.7 (1.1)	5.5 (0.8)	0.2 (0.6)	0.27	0.44	0.35
Leadership	5.6 (1.2)	5.5 (1.1)	0.1 (0.6)	0.72	0.67	1.00
Problem solving	5.2 (1.0)	5.2 (1.0)	0 (0.5)	1.00	0.59	0.35
Situational awareness	5.7 (0.9)	5.5 (0.8)	0.2 (0.8)	0.30	0.34	0.68
Resource utilization	5.8 (0.8)	5.8 (0.8)	0 (0.6)	1.00	1.00	N/A^##^
Communication	5.6 (1.2)	5.6 (1.2)	0 (0.6)	1.00	0.67	0.60
Average	5.6 (0.9)	5.5 (0.8)	0.1 (0.4)	0.44	0.38	1.00

Data are presented as mean (SD). P-value given for paired *t*-test.

3:1C:V: 3:1chest compression to ventilation ratio, CCaV: chest compression with asynchronous ventilations, CC + SI: chest compression with sustained inflation, CPR: cardiopulmonary resuscitation

^#^Standard: either 3:1C:V or CCaV.

^##^Identical scores (i.e., paired differences were all equal to 0), therefore unable to perform paired *t*-test.

### Qualitative results

Eight human factor themes were extracted from video analysis of 19 debrief sessions. These themes were subdivided into physical ergonomics (fatigue, position of team members), cognitive (familiarity, simplicity of ventilation, mental load, and timing), and teamwork/organizational ergonomics (communication, role switching, and shared mental load) sub-themes (Table 6). Additional themes included suggestions/concerns for implementation (skepticism, education for new technique, and improving timing) and simulation realism (Table 6). Participants were less familiar with CC + SI although many found it better for positioning, simpler to perform ventilation, better for role switching, and better for communication. Participants generally reported decreased mental load with CC + SI but had mixed feelings about timing and pacing with CC + SI and highlighted the necessity of a shared mental model for its success. Some participants found CC + SI more fatiguing compared to 3:1 C:V given its continuous CC without pause. Some participants were skeptical of CC + SI, and concern for possible increased pneumothoraxes. Participants also highlighted the need for education for successful implementation of CC + SI and that a two-person simulation was unrealistic for CPR.

**Table 6:** Thematic summary of debrief sessions with sample participant quotations.

Theme	Sub-Theme	Summary	Example Quote(s)
Physical Ergonomics	Fatigue	Continuous CC was more physically fatiguing than 3:1C:V	“[CC + SI was] more fatiguing… the little breath [with 3:1] gives your muscles a break.” (Interview #6)
	Position of Team Members	Easier for team members to access the patient	“I feel like you can fit more people… like your resuscitator doesn’t necessarily have to be at the head of the bed always and I feel like you could fit a compressor in more easily if you’re trying to do other things with the patient…” (Interview #19)
Cognition	Familiarity	CC + SI was less familiar and it would take time to get used to this new approach	“I think [CC + SI] went pretty well actually. Um, it just it felt weird… I could tell right away when we switched to our normal one, like, both of us, I think, were like much more confident.” *(Interview #1)*
	Simplicity of Ventilation	The airway role was easier to perform with CC + SI	“Honestly, from an airway perspective, [CC + SI vs PALS] was easier.” *(Interview #11)*
	Mental Load	Generally, decreased cognitive load required to perform CC + SI, resulting in more capacity to work through possible causes and treatments of cardiac arrest	“It felt much more mentally freeing… think about what might be going on or something… I only had to focus timing-wise… you didn’t have to synchronize so this would probably be easier.” *(Interview #6)*
	Timing	Timing and coordination were typically easier with CC + SI but some participants found it difficult to keep track of timing or pacing while providing CC during CC + SI	“I think it’s easier with sustained…because you’re not timing it. So just that little bit easier maybe for coordinating.” *(Interview #13)* “So, I think that one the challenge with [CC + SI], is keeping track of the compressions… so I kind of lost track of timing, like how long we’ve been doing it. The timer was different than the clock so I didn’t know how long we were on sustained inflations.” *(Interview #4)*
Teamwork/Organizational Ergonomics	Communication	Increased capacity to talk during CC + SI as not counting CC and ventilations out loud	“It’s potentially easier to communicate when you’re not always saying ‘1 and 2 and 3 and breath’… she was just doing continuous compressions so it was easier for me to just say ‘you’re okay to take airway?’ So, it was just easier to have a conversation.” *(Interview #10)*
	Role Switching	Switching roles (switching between performing CC and airway support) was easier to coordinate with CC + SI	“I thought it was more awkward when we switched [roles during] the normal way.” *(Interview #1)* “I thought [CC + SI] was easy. It was easy to switch [roles].” *(Interview #17)*
	Shared Mental Model	CC:SI might make it easier for team members to have a shared mental model, which is needed for success	“… this was nice because literally anyone who comes in to switch out… press and hold sustained, no confusion.” (Interview #11) “Make sure your leader is clear with what they want… Having a leader and a debrief, of course, if we time for all of that, to say ‘this is what we’re going to do’. I think it’s all about communicating.” (Interview #13)
Suggestions/Concerns for Implementation	Skepticism	Concern about increased risk of pneumothorax if performing CC + SI clinically	“My concern [about CC + SI] would be the peak pressures [and causing] a pneumothorax…” *(Interview #9)*
	Education for new technique	Education will be needed	“Getting everybody across the board on the same page… so many people.” (*Interview #10*)
	Improving Timing	Timing would be easier if it was 30 s per entire cycle	“29 s and 1 off [versus 30 s and 1 off… would be much easier]” (*Interview #18*)
Simulation Realism	Realism	A two-person team was not realistic for cardiac arrest	“…we would never compress for that long without help… am I secretly supposed to be doing something else? (*Interview #3*)

CC: chest compression, CC + SI: chest compression with sustained inflation

Participants were also asked for non-structured feedback. They provided suggestions to improve CC + SI for clinical implementation. Suggestions included: i) adjusting the timing of the first SI to allow subsequent SIs to “sync up” with 30 s intervals on the timer, ii) having a timer that buzzes or dings every 30 s to allow the person providing ventilation to focus less directly on the clock.

## Discussion

In this study, the human factors involved in CC + SI were compared to those for the current standard of care (either 3:1C:V or CCaV) by three methods including NASA-TLX score, CRM score, and thematic analysis of debrief sessions. The results can be summarized as follows: i) workload assessed with NASA-TLX was not different between CC + SI and standard CPR (3:1 C:V and CCaV); ii) no difference in team performance assessed using the OGRS with CC + SI compared to 3:1 C:V/CCaV; and iii) thematic analysis revealed participants were unfamiliar with CC + SI but generally found it easier to perform and better for communication compared to 3:1 C:V/CCaV.

The increase in the NASA-TLX raw score with the second simulation compared to the first simulation is due to the back-to-back nature of the cross-over study without a washout/rest period between simulations. Median (IQR) paired difference scores for both mental (−0.5 (−3,1), *p* = 0.02) and frustration (0 (−3,0), *p* = 0.02) dimensions increased over time (i.e., there was a higher perceived workload and frustration with the second simulation). When accounting for the crossover effect, there was only a significant difference in the temporal dimension (*p* = 0.005), as well as a difference in effort nearing significance (*p* = 0.07). Therefore, when participants performed CC + SI first, they had a degradation in perception of timing for the second CPR event and perceived increased effort/fatigue with the second CPR event. Li *et al* similarly reported increased fatigue with a decrease in peak pressures and CC depth during 3:1 C:V and CCaV over time.^15^

By NASA-TLX raw scores, overall workload was not increased with CC + SI compared to standard CPR. Further, CRM performance, based on the OGRS score, was not different with CC + SI than with standard CPR. These similarities are despite participants being trained in NRP +/- PALS, and many having significant experience with at least one of the latter two techniques. Participants performed CC a mean (SD) of 2.7 (2.3) times in the previous year and worked a mean (SD) of 8.9 (8.1) years in a NICU. Further, unfamiliarity with CC + SI was highlighted by multiple HCPs during the debrief session. Despite lack of familiarity and limited five minutes of teaching for CC + SI, CC + SI was generally reported to be an easier technique to perform, with easier communication, and switching roles than the standard CPR techniques. Most participants made positive comments about CC + SI.

Due to lack of evidence, AHA resuscitation guidelines do not provide recommendations for when to switch from the neonatal to the paediatric approach to CPR.^2^, ^3^ One NICU reported assigning patients to either 3:1 C:V or CCaV based on patient-specific criteria.^16^ Even with patient-specific criteria, using both CPR approaches in a unit may still lead to confusion about which approach to use for a given patient.^4^ For simplicity, it would therefore be ideal to have a single method of CPR that applies to all patients in the NICU. CC + SI has a shorter time to ROSC compared to 3:1 C:V in the delivery room,^9^, ^10^ and reduced time to ROSC and increased survival in paediatric piglets compared to CCaV.^6^, ^17^, ^18^ If CC + SI is superior to, or at least as good as 3:1 C:V and CCaV in neonates and infants, its implementation could potentially reduce confusion and simplify the approach to CPR.

Although the human factors, workload and team performance were no different compared to 3:1 C:V and CCaV, they can still be optimized for clinical implementation. Suggestions from participants included simplifying timing of SIs by implementing a beeping timer at 30 sec intervals, placing a visible timer near the bedside, or assigning a person to timing on a large enough team. Participants highlighted possible barriers to implementation including the cost and difficulty with training large numbers of HCPs to a new resuscitation technique and getting “buy-in” from HCPs to perform the new technique despite any perceived concerns such as risk of pneumothorax development/difficulty for recognition, which has not been reported in any of the completed trials.

There were limitations to our study. One limitation is the back-to-back timing of the two simulations each HCP performed. Ideally, a washout period between simulations would allow HCPs to full recover, but this was not feasible, however, we accounted for it in our statistical analysis. We did not run a teaching session on 3:1 C:V or CCaV prior to the simulation, but allowed participants to practice the technique and ask any questions prior to starting the simulation. Allowing participants to select CCaV or 3:1 C:V resulted in skewed distribution, although these were combined for statistical analysis. Some participants may have also benefited from brief teaching as a reminder, even if they did not have any specific questions. A further limitation included having only a single author calculate the OGRS score, who was not specifically trained in OGRS scoring which limited both the ability to assess inter-rater reliability and to ensure validity. Finally, the PremieHal manikin may have underestimated workload required for larger infants, which may be more relevant for CC + SI and CCaV than for 3:1 C:V.

## Conclusions

Chest compression with sustained inflation (CC + SI) is not more physically or cognitively demanding compared to the current standards of practice for neonatal and paediatric CPR. CC + SI appears to be feasible to be implemented for neonates and infant CPR. Further work is needed to assess the human factors and team dynamics for CC + SI in older paediatric patients prior to implementation.

## Author’s contribution

Conception and design: GMS, CM, BL, JD. Collection and assembly of data: CM, BL. Analysis and interpretation of the data: CM, BL, GMS. Drafting of the article: GMS, CM, BL, JD. Critical revision of the article for important intellectual content: GMS, CM, BL, JD. Final approval of the article: GMS, CM, BL, JD.

## CRediT authorship contribution statement

**Chelsea M.D. Morin:** Writing – review & editing, Writing – original draft, Resources, Project administration, Methodology, Investigation, Formal analysis, Data curation, Conceptualization. **Brenda H.Y. Law:** Writing – review & editing, Writing – original draft, Supervision, Resources, Project administration, Methodology, Investigation, Formal analysis, Data curation, Conceptualization. **Jonathan P. Duff:** Writing – review & editing, Writing – original draft, Supervision, Resources, Project administration, Methodology, Investigation, Formal analysis, Data curation, Conceptualization. **Georg M. Schmölzer:** Writing – review & editing, Writing – original draft, Supervision, Resources, Project administration, Methodology, Investigation, Formal analysis, Data curation, Conceptualization.

## Declaration of competing interest

The authors declare that they have no known competing financial interests or personal relationships that could have appeared to influence the work reported in this paper.
