# Supplementary material for: Assessing the human factors involved in chest compression with superimposed sustained inflation during neonatal and paediatric resuscitation: A randomized crossover study
Source: Resusc Plus. 2024 Jul 17;19:100721. doi: 10.1016/j.resplu.2024.100721 (PMC11301379; doi:10.1016/j.resplu.2024.100721)
Supplement: Supplementary Data 1 [file mmc1.docx]

**Appendix**

Scenario 1: A 1-month-old 3.5kg infant with profound sepsis/septic shock presented to the emergency department. They were given antibiotics, a fluid bolus, and started on maintenance fluid. They were intubated for apneic events and just arrived in the NICU for admission. Shortly after arrival to the NICU, the infant’s heart rate and oxygen saturations start to drop and they become severely bradycardic with heart rate in the 40’s.

Scenario 2: A 6-week-old 4kg infant with hypovolemic shock, thought to be secondary to a viral gastroenteritis, presented to the emergency department. They were given a fluid bolus and a second fluid bolus is being administered presently. They were intubated for apneic events and just arrived in the NICU for admission. Shortly after arrival to the NICU, the infant’s heart rate and oxygen saturations start to drop and soon after, they go into asystole.
