# Supplementary material for: Assessing the human factors involved in chest compression with superimposed sustained inflation during neonatal and paediatric resuscitation: A randomized crossover study
Source: Resusc Plus. 2024 Jul 17;19:100721. doi: 10.1016/j.resplu.2024.100721 (PMC11301379; doi:10.1016/j.resplu.2024.100721)
Supplement: Supplementary Data 3 [file mmc3.docx]

**Assessing the human factors involved in chest compression with superimposed sustained inflation during neonatal and pediatric resuscitation: a randomized crossover study protocol**

Chelsea MD Morin MD^12^, Brenda HY Law MD, MSc^12^, Jonathan Duff MD, MEd^2^, Georg M Schmölzer MD, PhD^12^

^1^Centre for the Studies of Asphyxia and Resuscitation, Neonatal Research Unit, Royal Alexandra Hospital, Edmonton, Alberta, Canada

^2^Department of Pediatrics, Faculty of Medicine and Dentistry, University of Alberta, Edmonton, Alberta, Canada

**Background**

Cardiopulmonary resuscitation (CPR), defined as chest compression (CC) with or without administration of vasopressor, is uncommon in children. CPR is performed in only about 1.1% of all neonatal intensive care unit (NICU) admissions^1^ and 1.4% of all pediatric intensive care unit (PICU) admissions^2^. Further, health care providers in the pediatric emergency department (PED) only perform a mean of 3 minutes of CC per year^3^.

Neonatal resuscitation guidelines differ from pediatric resuscitation guidelines. Neonatal resuscitation guidelines recommended 3:1 CC to ventilation ratio (C:V), and with a total event rate (ie. CC + ventilations) of 120/min^4^. Pediatric resuscitation guidelines recommend 30:2 C:V prior to establishment of an alternate airway and continuous CC with asynchronous ventilations (CCaV) with 120 CC/min and 20-30 ventilations/min after establishment of an alternate airway^5^. Both algorithms recommend optimizing ventilation first for severe bradycardia <60 and only starting chest compressions if heart rate is unresponsive to optimized ventilation^4,5^. Neonatal resuscitation guidelines suggest this same approach for asystole, but pediatric resuscitation guidelines recommend starting CC immediately for asystole^4,5^. The specific CPR algorithm followed depends on patient age, health care provider comfort, and even location of CPR (i.e., NICU, PICU, PED), especially for young infants as there are no specific guidelines for timing of transitioning from neonatal to pediatric resuscitation guidelines.

A newer technique for CPR, called chest compression with sustained inflation (CC+SI) may be beneficial for resuscitation in both neonates and pediatric patients^6,7^. Studies have shown reduced time to return of spontaneous circulation (ROSC) in neonatal piglets^6,8,9^, pediatric piglets^7^, and neonates^10^ as well as increased rate of ROSC in neonatal^6^ and pediatric piglets^7^. With this technique a sustained high-pressure inflation is provided simultaneously with CC^6^. Ventilation occurs passively, due to pressure changes caused by compression and recoil of the chest during CC. This technique not only has the potential to improve patient outcomes, but also to simplify resuscitation algorithms for neonates and pediatric patients. One barrier to studying CC+SI in pediatric patients is testing CC+SI in clinical simulations.

Clinical testing of novel CPR techniques is limited by the rarity of clinical events needing CPR. In addition to the potential physiological differences of CC+SI vs 3:1 C:V and CCaV, the clinical effectiveness of CC+SI might also be influenced by its effects on human factors such as physical ergonomics (force applied, physical fatigue, physical coordination with ventilation, equipment requirements), cognition (continuous chest compression might alter time keeping as there are no longer “cycles”), and team functioning (continuous chest compressions may alter how team members coordinate with each other). Understanding how a novel resuscitation technique affects human factors and team performance may be critical to facilitate the success of a novel intervention. Simulations have been successfully used to study physical ergonomics of chest compressions but have not been used to study team-based performance of CC+SI. Given the rarity and diversity of CPR events in pediatrics, team-based simulations may be more appropriate to evaluate these considerations, allowing for repeated, controlled, and standardized scenarios.

*Study Aim*

To our knowledge, there are no studies assessing the human factors involved in neonatal or pediatric resuscitation involving CC+SI. The aims of this study are to use standardized team-based simulations to 1) evaluate physical, cognitive, and team-based human factors of CC+SI in neonatal resuscitation teams as compared to standard 3:1 C:V and CCaV, 2) uncover potential barriers for implementing CC+SI in diverse clinical environments, and 3) compare human factors challenges of a novel CPR technique from different teams and physical environments.

*Hypotheses*

Primary hypothesis

We hypothesize that the physical, cognitive, and team-based human factors of performing CC+SI will be perceived as either equivalent to or less cognitively demanding than that of 3:1 C:V and CCaV.

Secondary hypotheses:

We hypothesize that there will be no significant barriers to implementation of CC+SI in diverse clinical environments. We also hypothesize that there will be some unforeseen human factors challenges with CC+SI within different teams and environments, and that our study will both highlight these challenges and provide multiple perspectives and ideas for improvements.

**Methods**

*Study Design*

This will be a cross-over randomized study with participants from two locations within the Stollery Children’s Hospital. The first is a 69-bed level 3 NICU located at the Royal Alexandra Hospital attached to the women’s hospital where high-risk deliveries occur (NICU-1). The second is an 18-bed level 4 medical/surgical/cardiac NICU at the University of Alberta Hospital (NICU-2).

The study will address the following PICOS question:

P: Healthcare professionals within the Stollery Children’s Hospital’s NICU-1 and NICU-2 including physicians, respiratory therapists, nurse practitioners, and nurses in teams of 2

I: basic simulation requiring CC, using CC+SI resuscitation technique, following a 5-minute CC+SI orientation

C: basic simulation requiring CC, using Pediatric Advanced Life Saving (PALS) or Neonatal Resuscitation Program (NRP) technique, as determined by the healthcare professionals

O: CC quality, cognitive load of CC+SI, ability to switch between roles with CC+SI

S: Crossover design with 10 NICU-1 and 10 NICU-2 simulations with 2 participants per simulation

*Sample Size*

We will aim to run 10 simulations in each of NICU-1 and NICU-2. Each simulation will require 2 healthcare worker participants to carry out the roles of ventilation and CC, therefore totaling 20 participants at each location, and 40 participants overall. There are no previous studies to base sample size on, but this sample size should be sufficient to allow for participation from a variety of disciplines within each location.

*Recruitment*

We will aim to recruit 40 healthcare works, 20 from each of NICU-1 and NICU-2 in order to run 10 simulations in each location, as above.

*Inclusion Criteria*

Healthcare professionals who have completed and been certified with NRP and/or PALS training within the last 2 years will be eligible for inclusion. Eligible healthcare professionals may include physicians (pediatricians, neonatologists, and NICU sub-specialty residents); nurses (registered nurses or licensed practical nurses); nurse practitioners, and respiratory therapists.

*Exclusion Criteria*

Healthcare professionals who do not consent to participate will be excluded. Healthcare professionals who have self-reported physical barriers to performing CC or CPR will also be excluded.

*Statistical Analysis*

Normally distributed data will be presented as mean (standard deviation) and skewed data will be presented as median (interquartile range). Paired Student’s t-test will be used for data comparison for normally distributed paired data and Wilcoxon matched-pairs signed-rank test for skewed paired data. Wilcoxon rank and repeated measures ANOVA were used to account for cross-over effect. Anonymized data will be input into a Redcap database and data will be assessed using STATA® software.

*Experimental Design and Randomization*

The simulation will be run with 2 scenarios, using a 2-step computer-generated randomizer (www.randomizer.org) to first randomize the order of the scenarios and second, randomize the order of resuscitation technique (ie. CC+SI or NRP/PALS). Block randomization will be used with blocks of 5, to allow for 2 blocks per location. Allocation concealment will be achieved using numbered, sealed, envelopes which will be opened just prior to commencing the first simulation. Blinding of the scenario and intervention will not be possible, as the participants will be performing the intervention for the given scenario and then filling out the post-simulation questionnaire and a researcher will be running the simulation. The results will be anonymized when they are input into the Redcap database to ensure blinding during statistical analysis.

After consent is obtained (see consent form attached), there will be a 5-minute teaching session (verbal explanation) to explain CC+SI and orient participants to the simulation manikin as well as to answer any questions they have.

The scenarios are as follows:

Scenario 1: A 1-month-old 3.5kg infant with profound sepsis/septic shock presented to the emergency department. They were given antibiotics, a fluid bolus, and started on maintenance fluid. They were intubated for apneic events and just arrived in the NICU for admission. Shortly after arrival to the NICU, the infant’s heart rate and oxygen saturations start to drop and they become severely bradycardic with heart rate in the 40’s.

Scenario 2: A 6-week-old 4kg infant with hypovolemic shock, thought to be secondary to a viral gastroenteritis, presented to the emergency department. They were given a fluid bolus and a second fluid bolus is being administered presently. They were intubated for apneic events and just arrived in the NICU for admission. Shortly after arrival to the NICU, the infant’s heart rate and oxygen saturations start to drop and soon after, they go into asystole.

Physical exam findings will be provided upon request (e.g. no palpable pulses, no heartbeat auscultated, equal air entry with ventilations). Both scenarios will end after 4 minutes, to allow time for the healthcare professionals to switch roles, but to avoid additional steps of resuscitation. If additional help, defibrillation pads, blood work, medications etc. are requested, the researcher running the simulations will redirect the team and explain that help is on the way, or that someone is working on their request (e.g. drawing up epinephrine). The simulation will end before the requested help, equipment, and/or medications arrive.

*Study Procedure*

The PremieHal (Gaumard, Miami, USA) manikin will be used for every simulation. This manikin has high-fidelity capabilities including the ability to produce visible cyanosis, palpable pulses, and lung and heart sounds that can be auscultated. Monitors (cardiac leads, oxygen saturation sensor, carbon dioxide sensor, and a blood pressure) will be connected to the manikin. Heart rate, blood pressure, oxygen saturations, respiratory rate, and temperature will all be visible for participants on the cardiorespiratory monitor. Gaumard software will be used to display vital signs for pre-built simulations which will be controlled from a Microsoft Surface laptop (Microsoft, Washington, USA).

Set-up specifics individualized based on location will include the following:

NICU-1: the simulation will take place in an empty patient room in NICU-1, as available. The infant will be placed in an isolette. A Neopuff^TM^ T-piece (Fisher & Paykel, Auckland, New Zealand) will both be available at the bedside as would be typical in NICU-1. Default settings on the Neopuff^TM^ will include a peak inspiratory pressure of 24 cm H_2_O and a peak end expiratory pressure of 5 cm H_2_O with a default flow rate of 10 L/min. The self-inflating bagger will also be set up with a default flow rate of 10 L/min.

NICU-2: the simulation will take place in either the simulation centre or an open patient room in NICU-2, as available. The infant will be placed in a Giraffe Warmer (GE Healthcare, Chicago, United States). A Neopuff^TM^ T-piece (Fisher & Paykel, Auckland, New Zealand) will both be available at the bedside as would be typical in NICU-2. Default settings on the Neopuff^TM^ will include a peak inspiratory pressure of 24 cm H_2_O and a peak end expiratory pressure of 5 cm H_2_O with a default flow rate of 10 L/min. The self-inflating bagger will also be set up with a default flow rate of 10 L/min.

The volunteers will be asked to fill out a post-resuscitation survey (see below) and will be given a gift card for $5 to Tim Hortons as thanks for their participation.

*Funding*

N/A. We have access to $5 Tim Hortons gift cards available to give to participants.

Post Simulation Questionnaire

Questionnaire to be completed on RedCap (QR code available) or on paper (paper copies available)

Demographics

Gender

What is your current role/position?

What field of pediatrics do you work in?

How many years have you worked in your current field?

Have you switched between your current field and another field in pediatrics? (eg. previously worked on an inpatient unit) If so, please explain.

Please state the approximate number of times you have performed CC in the last year (simulation or on a patient).

How often have you been team leader when CC were performed in the last year?

Request completion of Surgical TSX Load Index for each scenario.

Mental Demand: How mentally demanding was the task, on a scale of very low to very high?

Physical Demand: How physical demanding was the task, on a scale of very low to very high?

Temporal Demand: How hurried or rushed was the pace of the task, on a scale of very low to very high?

Performance: How successful were you in accomplishing what you were asked to do, on a scale of very low to very high?

Effort: How hard did you have to work to accomplish your level of performance, on a scale of very low to very high?

Frustration: How insecure, discouraged, irritated, stressed, and annoyed were you, on a scale of very low to very high?

Other questions

How difficult did you find learning chest compression with sustained inflation, on a scale of very low to very high?

How difficult did you find switching roles with sustained inflations, on a scale of very low to very high?

How difficult did you find switching roles with NRP/PALS, on a scale of very low to very high?

Please explain any challenges you found with performing chest compression with sustained inflation.

Please explain any barriers you foresee with implementing chest compression with sustained inflation.

**PARTICIPANT CONSENT FORM**

**Title of Study:** Assessing the human factors involved in chest compression with superimposed sustained inflation during neonatal and pediatric resuscitation: a randomized crossover study

Contact Information

Principal Investigator:

Chelsea Morin, MD

Centre for the Studies of Asphyxia and Resuscitation,

Neonatal Research Unit, Royal Alexandra Hospital,

10240 Kingsway Avenue NW, T5H 3V9

Edmonton, Alberta, Canada

Phone: +1 780 735 4660

Email: [Chelsea.morin@ualberta.ca](mailto:Chelsea.morin@ualberta.ca)

Supervisor:

Georg M. Schmölzer, MD, PhD

Centre for the Studies of Asphyxia and Resuscitation,

Neonatal Research Unit, Royal Alexandra Hospital,

10240 Kingsway Avenue NW, T5H 3V9

Edmonton, Alberta, Canada

Phone: +1 780 735 4660

Email: [georg.schmoelzer@me.com](mailto:georg.schmoelzer@me.com)

You are being invited to take part in a research study. Before you take part, a member of the study team is available to explain the project and you are free to ask any questions about anything you do not understand. You will be given a copy of this form for your records.

**Why am I being asked to take part in this research study?**

You are being asked to participate because you are a healthcare worker in the Pediatric ED, PICU, or NICU.

The goal of this study is to investigate the “human factors” associated with implementing a new chest compression technique.

**What is the reason for doing the study?**

Our research group, out of the Centre for the Studies of Asphyxia and Resuscitation, at the University of Alberta has developed a new resuscitation technique called “chest compressions with superimposed sustained inflations”. This technique has been studied extensively in animals, showing increased chances of survival and shorter duration of resuscitation. We are aiming to test this technique in basic resuscitation simulations and compare the “Human Factors” (eg. team dynamics, physical demands, cognitive demands) associated with this technique with standard resuscitation techniques.

**What will I be asked to do?**You will be asked to:

- Participate in a 5-minute overview of “chest compressions with sustained inflations”
- Participate on a 2-person team in 2 short simulations. One simulation will be run using your typical method of resuscitation, and the other using “chest compressions with sustained inflations”.
- Fill out a post-simulation questionnaire.
- The introduction, simulation, and questionnaire should take a total of approximately 15-20 minutes to complete.
- With your consent, the session with be recorded.
- With your consent, you will wear a stress sensor to record your heart rate and temperature.
- With your consent, study information will be stored in a secure data repository to facilitate future research.

**What are the risks and discomforts?**

The associated risks with this research may include physical discomfort from providing chest compressions. It is not possible to know all the risks that may happen in a study, but the researchers have taken all reasonable safeguards to minimize any known risks to a study participant.

**What are the benefits to me?**

While there may not be direct benefit to you, results from this study may help us optimize “chest compressions with sustained inflation” resuscitation which may allow us to save the lives of more children.

**Do I have to take part in the study?**

Being in this study is your choice. You do not have to answer any questions in the post-simulation survey that you are not comfortable with.

Due to the anonymous nature of survey participation, you can withdraw your data up until the time of survey submission. Simply do not submit your data and nothing will be inputted into the database and the video recording will be deleted. Once you have submitted your data it is not possible for it to be removed.

Your participation in this study is completely voluntary. Should you choose to participate in the research activity, you may decide at any time after you have begun, to withdraw.

**Will I be paid to be in the research?**

You will receive a $5 gift card for participating. If you choose to withdraw from the study partway through, you will not receive the gift card due to the limited number the study team has available.

**What happens if I am injured because of this research?**

If you become ill or injured as a result of being in this study, you will receive necessary medical treatment, at no additional cost to you. By signing this consent form, you are not giving up any of your legal rights or releasing the investigators, institution and/or sponsors from their legal and professional responsibilities.

**Will my information be kept private?**

During this study we will do everything we can to make sure that all information you provide is kept private. No information relating to this study that includes your name or video of yourself will be released outside of the researcher’s office or published by the researchers unless you give us your express permission. Sometimes, by law, we may have to release your information with your name so we cannot guarantee absolute privacy. However, we will make every legal effort to make sure that your information is kept private.

During research studies it is important that the data we get is accurate. For this reason, your data, including your name, may be looked at by people from the Research Ethics Board.

After the study is done, we will still need to securely store your data that was collected as part of the study. Surveys will be stored in the Centre for the Studies of Asphyxia and Resuscitation research office which is locked. Electronic data will be uploaded to and stored on the RedCap database which is password protected and video recordings will be encrypted and password protected. In keeping with University of Alberta standards, data will be stored for a minimum of 5 years after the end of the study.

**What if I have questions?**

If you have any questions about the research now or later, please contact Chelsea Morin at [Chelsea.morin@ualberta.ca](mailto:Chelsea.morin@ualberta.ca).

If you have any questions regarding your rights as a research participant, you may contact the University of Alberta Research Ethics Office at [reoffice@ualberta.ca](mailto:reoffice@ualberta.ca) or 780-492-2615 and quote Ethics ID Pro00130209. This office is independent of the study investigators.

The study is being supported by the Women and Children’s Research Institute, the Canadian Lung Association and the Canadian Institute for Health Research. The Institution and Principal Investigator are getting money from the study sponsor to cover the costs of doing this study. You are entitled to request any details concerning this compensation from the Principal Investigator.

**How do I indicate my agreement to be in this study?**

By signing below, you understand:

- That you have read the above information and have had anything that you do not understand explained to you to your satisfaction.
- That you will be taking part in a research study.
- That you may freely leave the research study at any time.
- That you do not waive your legal rights by being in the study
- That the legal and professional obligations of the investigators and involved institutions are not changed by your taking part in this study.
- That you agree to the data being stored as part of a data repository (where applicable)

**SIGNATURE OF STUDY PARTICIPANT**

_______________________________

Name of Participant

________________________________ _____________________

Signature of Participant Date

**SIGNATURE OF PERSON OBTAINING CONSENT**

________________________________ _____________________

Name of Person Obtaining Consent Contact Number

A copy of this consent form has been given to you to keep for your records and reference.
